# Impact of rural versus urban setting on kidney markers: a cross-sectional study in South-Kivu, DRCongo

**DOI:** 10.1186/s12882-021-02431-w

**Published:** 2021-06-25

**Authors:** Mannix Imani Masimango, Michel P. Hermans, Espoir Bwenge Malembaka, Pierre Wallemacq, Ernest Kiswaya Sumaili, Catherine Fillée, William D’Hoore, Cheryl A. Winkler, Sophie Limou, Michel Jadoul

**Affiliations:** 1 Department of Internal Medicine, Hôpital Provincial Général de Référence de Bukavu, Université Catholique de Bukavu, Bukavu, Democratic Republic of Congo; 2 Department of Nephrology, Cliniques Universitaires Saint-Luc, Université Catholique de Louvain, Brussels, Belgium; 3 Department of Endocrinology and Nutrition, Cliniques Universitaires Saint-Luc, Université Catholique de Louvain, Brussels, Belgium; 4 Faculté de Médecine, Ecole Régionale de Santé Publique, ERSP, Université Catholique de Bukavu, Bukavu, Democratic Republic of Congo; 5 Department of Epidemiology, Johns Hopkins Bloomberg School of Public Health, Johns Hopkins University, Baltimore, USA; 6 Department of Laboratory Medicine, Cliniques Universitaires Saint-Luc, Université Catholique de Louvain, Brussels, Belgium; 7 Department of Nephrology, Université de Kinshasa, Kinshasa, Democratic Republic of Congo; 8 Institute of Health and Society (IRSS), Université catholique de Louvain, Brussels, Belgium; 9 Basic Science Laboratory, Genetic Epidemiology Section, Frederick National Laboratory, Frederick, Maryland USA; 10 Centre de Recherche en Transplantation et Immunologie, Institute for Transplantation in Urology-Nephrology and Ecole Centrale de Nantes, Computer Sciences and Mathematics Department, Université de Nantes, Inserm, UMR1064, Nantes, France

**Keywords:** CKD screening tests, Proteinuria dipstick, Albumin-to-creatinine ratio, Rural-urban location, Prevalence, Performance, Determinants, DRCongo

## Abstract

**Background:**

Most studies of chronic kidney disease (CKD) in Sub-Saharan Africa (SSA) have been conducted in urban settings. They relied on GFR estimated from serum creatinine alone and on the inexpensive, convenient urinary dipstick to assess proteinuria. The dipstick for proteinuria has not been directly compared with the gold standard albumin-to-creatinine ratio (ACR) in a large-sized study in SSA. We hereby assessed the influence of rural versus urban location on the level, interpretation, and diagnostic performance of proteinuria dipstick versus ACR.

**Methods:**

In a cross-sectional population-based study of CKD in both urban (*n* = 587) and rural (*n* = 730) settings in South-Kivu, Democratic Republic of Congo (DRC), we assessed the prevalence, performance (sensitivity, specificity, positive predictive value and negative predictive value) and determinants of a positive dipstick proteinuria as compared with albuminuria (ACR). Albuminuria was subdivided into: A1 (< 30 mg/g creatinine), A2 (30 to 299 mg/g creatinine) and A3 (≥ 300 mg/g creatinine).

**Results:**

The overall prevalence of positive dipstick proteinuria (≥ 1+) was 9.6 % (95 % CI, 7.9–11.3) and was higher in rural than in urban residents (13.1 % vs. 4.8 %, *p* < 0.001), whereas the prevalence of albuminuria (A2 or A3) was similar in both sites (6 % rural vs. 7.6 % urban, *p* = 0.31). In both sites, dipstick proteinuria ≥ 1 + had a poor sensitivity (< 50 %) and positive predictive value (< 11 %) for the detection of A2 or A3. The negative predictive value was 95 %. Diabetes [aOR 6.12 (1.52–24.53)] was a significant predictor of A3 whereas alkaline [aOR 7.45 (3.28–16.93)] and diluted urine [aOR 2.19 (1.35–3.57)] were the main predictors of positive dipstick proteinuria.

**Conclusions:**

ACR and dipstick proteinuria have similar positivity rates in the urban site whereas, in the rural site, dipstick was 2-fold more often positive than ACR. The poor sensitivity and positive predictive value of the dipstick as compared with ACR makes it unattractive as a screening tool in community studies of CKD in SSA.

**Supplementary Information:**

The online version contains supplementary material available at 10.1186/s12882-021-02431-w.

## Introduction

Chronic kidney disease (CKD) is a global public health problem, particularly in sub-Saharan Africa (SSA) [1, 2]. Despite the rapid urbanization, SSA populations are still mostly rural [3], yet over 90 % of CKD studies have been conducted in urban settings [1]. In a meta-analysis, Stanifer et al. reported no difference in CKD prevalence between urban and rural settings [1]. However, recent data on CKD burden are sometimes conflicting. Two studies from Ghana and Tanzania [4, 5], have found CKD to be more prevalent in urban than rural populations, contrasting with opposite findings from Cameroon [6] and Malawi [7]. These discrepancies are not solely explained by the variable prevalence of classical risk factors of CKD (hypertension, diabetes, ageing, obesity) between rural and urban population [4, 6, 8, 9]. The widespread use of the proteinuria dipstick for CKD detection in SSA may also account for some differences in CKD estimates between rural and urban settings. In fact, it is known that the proteinuria dipstick has imperfect accuracy in the diagnosis of albuminuria, as assessed by albumin-creatinine ratio (ACR) [10–13]. We wanted to take advantage of the inclusion of both urban (*n* = 587) and rural subjects (*n* = 730) in our study to assess the performance of proteinuria dipstick compared to albuminuria in both settings. In addition, we hypothesized that substantial differences between urban and rural lifestyles (physical activity, diet; body composition) could impact the level and interpretation of kidney markers.

## Methods

### Study setting and population

The design and methods of the study have been published [8]. Participants were randomly selected from the general population of South-Kivu province, in the Eastern part of the DRC. In brief, a total of 1350 adults (≥ 18 years) from two locations (Ibanda, urban site and Katana, rural site) were contacted to participate to the study between October 2016 and April 2017. South Kivu had an estimated population of 6,742,196 inhabitants in 2017, distributed across 34 health zones (3 urban and 31 rural zones). Katana is a rural zone, located 45 km from Ibanda, a district of the cosmopolitan Bukavu city.

### Clinical measurements

Hypertension was defined as systolic blood pressure ≥ 140 mm Hg and/or diastolic blood pressure ≥ 90 mm Hg and/or self-reported use of antihypertensive medications [14]. Diabetes was defined as fasting glucose ≥ 126 mg/dL, postprandial glucose ≥ 200 mg/dL and/or self-declared diabetes treated with glucose-lowering agent(s) [15]. Body weight (Kg), height (cm), waist circumference (WC) (cm), hip circumference (HC) (cm), and mid-upper arm circumference (MUAC) (cm) were measured using WHO standard protocols and calibrated devices [16]. Obesity was defined as a BMI ≥ 30 kg/m^2^ [17]. Central obesity was defined WC ≥ 94 cm in males and ≥ 80 cm in females [18].

We measured visceral fat (% of body weight) and total body fat (% of body weight) by bioelectrical impedance analysis, using an OMRON BF508 body composition monitor (BF 508, OMRON Healthcare Co., Muko, Kyoto, 617-0002 JAPAN) at a fixed frequency of 50 kHz. The results provided by an OMRON device equipped with such a technology were shown to be well correlated (r^2^ = 92–96 %) with the gold standards (magnetic resonance imaging and Dual X-Ray Absorptiometry), both regarding fat mass and visceral fat [19]. Lean body mass (LBM) was calculated as the difference between body weight and total body fat mass. Skeletal muscle mass (SMM) was estimated according to the formula: SMM= (0.488 x LBM) − 2.22. This formula was established from a reference cohort of 185 subjects from sub-Saharan Africa [Benin (*n* = 110) and DRC (*n* = 75) of both sexes (83 men & 102 women)], who had simultaneous assessment by bioelectrical impedance analysis (OMRON Body Fat analyzer) of BMI, relative and total fat mass, and relative and total muscle mass, based on the linear relationship between whole-body SMM and fat-free mass present in both genders (Michel Hermans, unpublished).

### Laboratory measurements

Blood and random spot urine samples were collected at home from all participants. Tests for glycaemia and urine dipstick (Multistix 10 SG®, Siemens Healthcare Diagnostics, France) were immediately performed on site by the study team. Thereafter, the samples were stored (urine immediately, blood within 10 min) in an ice pack carrier, transported on the same day to the laboratory of Hôpital Provincial Général de Référence de Bukavu, centrifuged as appropriate and stored at -20 °C. Frozen serum and urine samples were sent to the Clinical Chemistry laboratory of the Cliniques Universitaires Saint-Luc (Brussels, Belgium), where samples were thawed prior to analysis. Serum creatinine (compensated Jaffe method, IDMS-traceable), urinary albumin and urinary creatinine were measured with a Roche Cobas analyzer (Roche Diagnostics, 8000, module c702, Rotkreuz, Switzerland). Serum cystatin C was measured using a PETIA method on the SPA PLUS® analyzer (Binding Site, Birmingham, UK). This method has been standardized according to the reference material ERM-DA471/IFCC. An elevated serum CRP level was defined as ≥ 3 mg/L [20].

### Assessment of kidney markers

The results of urine dipstick were read as negative/trace or positive at 1+, 2+, 3+, 4 + corresponding approximately to urinary protein concentrations < 30, 30, 100, 300 and ≥ 2000 mg/dL.

For the calculation of the prevalence of proteinuria by dipstick and its diagnostic value [sensitivity, specificity, positive predictive value (PPV), negative predictive value (NPV)] as compared with the gold standard albumin-creatinine ratio (ACR), we excluded subjects with urinary dipstick positive for blood and/or leucocytes and/or nitrites. Urine pH was categorized as < 6.5, 6.5-7 and ≥ 7.5. A pH ≥ 7.5 was considered as alkaline [21]. Urine specific gravity was categorized as < 1.010, 1.010–1.020 and > 1.020. Urine was considered as diluted if specific gravity was < 1.010. We measured urine osmolality in a random subgroup of rural (*n* = 50) and urban (*n* = 50) subjects (Arkray Inc, Osmo Station, OM-6060, Kyoto, Japan). Glomerular filtration rate (eGFR) was estimated from serum creatinine, serum cystatin C or both using the CKD-EPI equations, without correction for ethnicity [22]. CKD was defined by eGFR < 60 mL/1.73 m^2^ and/or ACR ≥ 30 mg/g [23].

### Statistical analyses

All analyses were performed using Stata, version 12.1 (StataCorp LP). Normally-distributed continuous variables are presented as means ± 1 standard deviation whereas non-normally distributed variables are presented as medians [interquartile range], and qualitative variables as crude counts and percentages. Difference in proportions and means were compared by Pearson’s chi-square test or Student’s *t* test, as appropriate. According to KDIGO [23], ACR was subdivided into three sub-categories: <30 mg/g creatinine (A1), 30 to 299 mg/g creatinine (A2) and ≥ 300 mg/g creatinine (A3), and compared with the results of the dipstick proteinuria on the same sample. The diagnostic value (sensitivity, specificity, positive predictive value, negative predictive value) of dipstick proteinuria 1 + or greater was compared with ACR A2 or A3 as the reference. Univariate and multivariable logistic regression analysis was used to identify factors independently associated with proteinuria ≥ 1 + by dipstick and with albuminuria (ACR ≥ 300 mg/g). We assessed the correlation between urine specific gravity and urine osmolality using a scatter plot with a linear regression line. A *p* value < 0.05 was considered as statistically significant.

## Results

### General characteristics of participants

After excluding pregnant women (*n* = 24) and subjects who declined to participate (*n* = 9), 1317 adults (730 in rural site and 587 in urban site) were included in the present study. Among 1296 of 1317 individuals who provided urine on site, 162 were excluded from further urine analyses because of positivity of urinary dipstick for blood and/or leucocytes and/or nitrites. A flow chart of the study population and laboratory measurements is depicted in supplementary Fig. 1. The sex distribution was similar between sites (*p* = 0.15). Compared to urban participants, rural individuals were older (44.6 years vs. 36.7 years, *P <* 0.001), less educated, and more likely to be farmers (71.1 % vs. 1.2 %, *p* < 0.001). The general characteristics of study participants are described in Table 1.

**Table 1:** General characteristics of the study population stratified by site and sex

Characteristics	Overall *n* = 1317	Rural *n* = 730	Urban *n* = 587	*P*-value	Male *n* = 515	Female *n* = 802	*P*-value
Age, years	41.1 ± 17.1	44.6 ± 17	36.7 ± 16.4	< 0.001	40.4 ± 17	42.1 ± 17.3	0.07
Female, n (%)	802 (60.9)	432 (59.3)	370 (63.3)	0.15			
**Occupation, n (%)**				< 0.05			< 0.05
Gov.employee/NGOs	123 (9.3)	57 (7.8)	66 (12.2)		86 (16.7)	37 (4.6)	
Farming	526 (40)	519 (71.1)	7 (1.2)		178 (34.6)	348 (43.4)	
Business	128 (9.8)	45 (6.2)	83 (14.2)		63 (12.2)	65 (8.1)	
None	540 (40)	109 (14.9)	431 (73.4)		188 (36.5)	352 (43.9)	
**Education level, n (%)**				< 0.05			< 0.05
None	483 (36.7)	395 (54.1)	88 (15)		125 (24.3)	358 (44.6)	
Primary	280 (21.3)	194 (26.6)	86 (14.7)		130 (25.2)	150 (18.7)	
Secondary	420 (31.9)	132 (18.1)	288 (49.1)		184 (35.7)	236 (29.4)	
Post-secondary	134 (10.2)	9 (1.2)	125 (21.3)		76 (14.8)	58 (7.2)	
**Marital status, n (%)**				< 0.05			
Married	938 (71.2)	576 (78.9)	362 (61.7)		387 (75.1)	551 (68.7)	< 0.05
Single	238 (18.1)	57(7.8)	181 (30. 8)		106 (20.6)	132 (16.5)	*NS*
Divorced/Widowed	125 (9.5)	84 (11.5)	41 (7)		11 (2.1)	114 (14.2)	< 0.05
Current smoking	71 (5.4)	53 (7.3)	18 (3.1)	< 0.05	47 (9.1)	24 (3)	< 0.05
Alcohol consumption	562 (42.7)	344 (47.1)	218 (37.1)	< 0.05	305 (59.2)	257 (32)	< 0.05
Use of medicinal plants	293 (22.2)	121 (16.6)	172 (29.3)	< 0.05	118 (22.9)	175 (21.8)	*NS*
Use of NSAIDs	435 (33)	212 (29)	223 (38)	< 0.05	166 (32.2)	269 (33.5)	*NS*
**Family history, n (%)**							
Diabetes	177 (13.4)	25 (3.4)	152 (25.9)	< 0.001	68 (13.2)	109 (13.5)	NS
Hypertension	347 (26.3)	109 (14.2)	238 (40.5)	< 0.001	116 (22.5)	231 (28.8)	0.012
**Personal history, n (%)**							
Hypertension	148 (11.2)	90 (12.3)	58(9.9)	0.17	39 (7.6)	109 (13.6)	0.001
Diabetes	36 (2.7)	8 (1.1)	28 (4.8)	< 0.001	16 (3.1)	20 (2.5)	NS

*NGOs *nongovernmental organizations, *NSAIDs* nonsteroidal anti-inflammatory drugs

### Clinical characteristics of participants by site and sex

The clinical characteristics of participants stratified by site and sex are provided in Table 2. Urban participants had higher BMI (24.8 ± 4.9 vs. 22.1 ± 3.4 kg/m^2^, *p* < 0.001), WC (85.2 ± 13.5 vs. 80.1 ± 9.9 cm, *p* < 0.001), HC (93.5 ± 12.7vs 89.1 ± 9.1 cm, *p* < 0.001), MUAC (27.9 ± 3.7 vs. 25.8 ± 2.8 cm, *p* < 0.001), relative body fat (30.7 ± 12.7 % vs. 24.1 ± 11.03 %, *p* < 0.001) and visceral fat (6.3 ± 3.6 % vs. 5.0 ± 2.7 %) than rural participants. In contrast, rural participants had significantly higher relative skeletal muscle mass (33 ± 5.1 % vs. 30.2 ± 5.8 %, *p* < 0.001) than urban subjects.

**Table 2:** Clinical characteristics of the study population stratified by site and sex

Characteristics	Overall *n* = 1317	Rural *n* = 730	Urban *n* = 587	*P*-value	Male *n* = 515	Female *n* = 802	*P*-value
Systolic blood pressure (mmHg)	122 ± 20.7	121.5 ± 20.5	123.3 ± 21.0	0.28	124.3 ± 18.4	120.6 ± 22	0.002
Diastolic blood pressure (mmHg)	78.8 ± 11.8	77.5 ± 11.6	80.4 ± 12.0	< 0.001	78.9 ± 11.4	78.7 ± 12.2	0.78
Weight (kg)	59.7 ± 12.4	56.1 ± 9.8	64.0 ± 13.9	< 0.001	59.9 ± 10.1	59.5 ± 13.7	0.57
Height (cm)	160.2 ± 8.5	159.6 ± 8.4	160.9 ± 8.6	0.03	165.5 ± 8	157.0 ± 7.2	< 0.001
BMI (Kg/m^2^)	23.3 ± 4.3	22.1 ± 3.4	24.8 ± 4.9	< 0.001	22 ± 3	24.1 ± 4.8	< 0.001
BMI ≥ 30 n, (%)	115 (8. 9)	21 (3)	94 (16.3)	< 0.001	12 (2.4)	103 (13)	< 0.001
Waist circumference (cm)	82.4 ± 11.9	80.1 ± 9.9	85.2 ± 13.5	< 0.0001	79.7 ± 9.4	84.1 ± 12.9	< 0.001
Enlarged WC^a^ n, (%)	524 (39.9)	243 (33.3)	281 (47.8)	< 0.001	48 (2.3)	476(59.4)	< 0.001
Hip circumference (cm)	91.1 ± 11.1	89.1 ± 9.1	93.5 ± 12.7	< 0.0001	87.4 ± 8.7	93.4 ± 11.8	< 0.001
Abnormal WHR^b^ n, (%)	898 (68.2)	462 (63.3)	340 (57.9)	< 0.001	281 (54.6)	617 (76.9)	< 0.001
MUAC (cm)	26.8 ± 3.4	25.8 ± 2.8	27.9 ± 3.7	< 0.0001	26.4 ± 2.8	27.0 ± 3.7	0.001
Body Fat (%)	27 ± 12.2	24.1 ± 11.03	30.7 ± 12.7	< 0.0001	16.8 ± 8.0	33.3 ± 10	< 0.001
Visceral fat (%)	5.6 ± 3.2	5.0 ± 2.7	6.3 ± 3.6	< 0.001	5.3 ± 3.5	5.7 ± 3	0.03
Skeletal muscle mass (%)	31.7 ± 5.6	33 ± 5.1	30.2 ± 5.8	< 0.001	36.8 ± 3.6	28.6 ± 4.2	< 0.001
Hypertension, n (%)	266 (20.2)	134 (18.4)	132 (22.5)	0.06	102 (19.8)	164 (20.4)	0.77
Diabetes, n (%)	57 (4.3)	21 (2.9)	36 (6.1)	0.004	24 (4.7)	33 (4.1)	0.67
HIV infection, n (%)	5 (0.4)	1(0.1)	4(0.8)	0.96	3(0.6)	2 (0.3)	0.34

*BMI* Body Mass Index, *WHR* waist-hip ratio, *MUAC* Mid-upper arm-circumference, *HIV *Human Immunodeficiency Virus

^a^Enlarged WC: ≥94 cm in men, ≥ 80 in women

^b^Abnormal WHR: ≥ 0.90 for men, ≥ 0.85 for women

As expected, male subjects had higher relative skeletal muscle (37 ± 4 % vs. 29 ± 4 %, *p* < 0.001) than women, whereas women showed higher WC (84.1 ± 12.9 vs. 79.7 ± 9.4 cm), HC (93.4 ± 11.8 vs. 87.4 ± 8.7 cm, *p* < 0.001), MUAC (27.0 ± 3.7 vs. 26.4 ± 2.8 cm), BMI (24.1 ± 4.8 vs. 22 ± 3 kg/m^2^), relative body fat (33.3 ± 10 vs. 16.8 ± 8.0 %) and visceral fat (5.7 ± 3 vs. 5.3 ± 3.5 %) than men (all, *p* < 0.05).

Regarding comorbidities, urban residents were more likely to have obesity (16.3 % vs. 3 %), hypertension (22.5 % vs. 18.4 %) and diabetes (6.1 % vs. 2.9 %), whereas HIV prevalence (0.4 %) did not differ by site.

### Biological characteristics of participants

Table 3 compares the laboratory data of urban and rural participants. The prevalence of dipstick proteinuria (≥ 1+) was 9.6 % (95 % CI, 7.9–11.3), and much higher in rural than in urban residents (13.1 % vs. 4.8 %, *p* < 0.001). Compared to urban participants, the urine of rural participants was much more likely to be alkaline (pH ≥ 7.5) (55.9 % vs. 25.1 %, *p* < 0.001) and diluted (69.9 % vs. 30.1 %, < 0.001). Urine osmolality did not differ significantly by site (768 ± 216 urban vs. 673 ± 298 rural, *p* = 0.071). Moreover, urinary osmolality did not correlate significantly with urine specific gravity, neither in the whole group (*r* = 0.138, *p* = 0.177), nor in the rural (*r* = -0.0099, *p* = 0.947) and urban (*r* = 0.224, *p* = 0.117) subgroups (Supplementary Fig. 2).

**Table 3:** Biological characteristics of the study population stratified by site and sex

Characteristics	Overall *n* = 1317	Rural *n* = 730	Urban *n* = 587	*P*-value	Male *n* = 515	Female n = 802	*P*-value
CRP (mg/L), n (%)							0.74
< 3	883 (73)	485 (70.7)	398 (76.1)	0.036	343 (72.5)	540 (73.4)	
≥ 3	326 (27)	201 (29.3)	125 (23.9)		130 (27.5)	196(26.3)	
TSH (mU/L)	1.48 (1-2.2)	1.47 (0.9–2.3)	1.48 (1.01-2)		1.6 (1.07–2.45*)*	1.39 (0.92–1.98)	
**Kidney markers**							
Serum creatinine (mg/dL)	0.89 ± 0.49	0.87 ± 0.49	0.92 ± 0.49	0.07	0.98 ± 0.51	0.83 ± 0.46	< 0.001
Serum cystatin C (mg/L)	0.93 ± 0.28	0.93 ± 0.28	0.93 ± 0.29	0.49	0.96 ± 0.31	0.90 ± 0.27	< 0.001
eGFR (mL/min/1.73 m^2^)							
CKD-EPI creatinine	94.6 ± 22.7	94.8 ± 22.8	94.4 ± 22.7	0.77	97.3 ± 22.2	92.8 ± 22.9	< 0.001
CKD-EPI cystatin C	92.6 ± 21.1	91.5 ± 21.1	94.1 ± 21.1	0.03	92.1 ± 21.1	92.9 ± 21.1	0.54
CKD-EPI combined	100.4 ± 21.6	100.1 ± 21.7	100.9 ± 21.6	0.51	101.9 ± 21.2	99.4 ± 21.9	0.05
**eGFR < 60 mL/1.73m**^**2**^**), n (%)**							
CKD-EPI creatinine	67 (5.43)	34 (4.89)	33 (6.12)	0.34	17 (3.48)	50 (6.7)	0.014
CKD-EPI cystatin C	80 (6.73)	48 (7.06)	32 (6.29)	0.599	30 (6.42)	50 (6.93)	0.736
CKD-EPI combined	56 (4.72)	27 (3.97)	29 (5.73)	0.157	18 (3.85)	38 (5.29)	0.256
Urine Dipstick analysis							
**Dipstick proteinuria** ≥ 1 +	109 (9.6)	86 (13.1)	23 (4.8)	< 0.001	53 (11.3)	56 (8.4)	0.10
**pH, n(%)**							
< 6.5	360 (27.8)	118 (16.41)	242 (41.9)	< 0.001	130 (25.64)	230 (29.11)	0.078
6.5-7.0	390 (30.1)	199 (27.7)	191 (33.1)	0.02	150 (29.59)	240 (30.38)	0.762
≥ 7.5	547 (42.2)	402 (55.9)	145 (25.1)	< 0.001	227 (44.77)	320 (40.51)	0.129
**Specific gravity,n(%)**				< 0.001			0.024
< 1.010	191 (14.7)	148 (20.6)	43 (7.5)		71 (14.7)	120 (15.2)	
1.010–1.020	737 (56.8)	446 (61.9)	291 (50.4)		311 (61.3)	426 (53.9)	
>1.020	369 (28.4)	126 (17.5)	243 (42.1)		125 (24.6)	244 (30.9)	
**Quantitative urinalysis**							
Urine creatinine, mg/L (IQR)	156 (100–226)	147(91.7-214.7)	178 (124–240)		173(106–246)	149 (97–113)	
Urine albumin, mg/L (IQR)	5.4 (2.4–12.4)	4.9 (2.3–11.3)	6 (2.8–13)		4.2 (2.1–9.5)	6.1(2.9–13.6)	
uACR ,mg/g (IQR)	3.5 (2.0-7.2)	3.7(2.1–7.3)	3.2 (1.8–7.1)		2.7 (1.6–5.4)	4.1 (1.5–8.2)	
ACR ≥ 30 mg/g, *n = 1070*	71 (6.6)	38 (6)	33 (7.6)	0.31	25 (5.9)	46 (7.1)	0.431

*CRP *C-Reactive Protein, *TSH* thyroid stimulating hormone, *eGFR* estimated glomerular filtration rate, *CKD-EPI*, chronic kidney disease epidemiology equation, *IQR* interquartile range

The prevalence of significant albuminuria (A2 or A3) was 6.6 % (95 % CI, 5.1–8.1) without difference by site.

Serum creatinine (0.92 ± 0.49 urban vs. 0.87 ± 0.49 mg/dL rural, *p* = 0.07) and cystatin C (0.93 ± 0.29 urban vs. 0.93 ± 0.28 mg/L rural, *p* = 0.49) levels did not differ by site. eGFRcys was higher in urban than rural participants (94.1 ± 21.1 vs. 91.5 ± 21.1 mL/min/1.73m^2^, *p* = 0.03), whereas eGFRcr did not differ by site. Both serum creatinine (0.98 ± 0.51 vs.0.83 ± 0.46 mg/dL, *p* < 0.001) and cystatin C (0.96 ± 0.31 vs. 0.90 ± 0.27 mg/L, *p* < 0.001) was significantly higher in men than in women.

### Comparison between dipstick and ACR in the study population

Supplementary Tables S1 and S2 show the positivity rates of dipstick proteinuria by ACR category (A1, A2, A3) and the diagnostic performance of dipstick proteinuria to detect albuminuria of grade A2 or more in the whole group (a), and in the urban (b) and rural (c) sites.

In comparison with A2 or more, dipstick proteinuria had an overall sensitivity, specificity, PPV and NPV of 19.2 %, 90.1 %, 10.2 and 95.0 %, respectively. In the rural site, the sensitivity and specificity of dipstick proteinuria (≥ 1+) were 17.2 and 86.7 %, respectively, whereas in the urban site, the sensitivity and specificity of dipstick proteinuria (≥ 1+) were 21.7 and 95.6 %, respectively.

In comparison with A3, dipstick proteinuria had an overall sensitivity, specificity, PPV and NPV of 30.8 %, 89.9 %, 4.1 and 98.9 %, respectively. In the rural site, the sensitivity and specificity of dipstick proteinuria (≥ 1+) were 75 and 86.9 %, respectively, whereas in the urban site, the sensitivity and specificity of dipstick proteinuria (≥ 1+) were 11.1 and 94.6 %, respectively.

### Predictors of proteinuria (dipstick ≥ 1+) and ACR ≥ 300 mg/g by logistic regression

In univariate logistic regression, rural residency [uOR 2.98 (1.85–4.80)], alkaline urine [uOR 8.76 (4.35–17.61)] and diluted urine [uOR 3.90 (2.52–6.04)] were the main predictors of dipstick proteinuria. In multivariable logistic regression, alkaline urine [aOR 7.45 (3.28–16.93)] and diluted urine [aOR2.19 (1.35–3.57)] were the main predictors of dipstick proteinuria, whereas only diabetes [aOR 6.12 (1.52–24.53)] was a significant predictor of ACR ≥ 300 mg/g (but not of positive dipstick proteinuria) (Table 4).

**Table 4:** Univariate and multivariable logistic regression of factors associated with dipstick proteinuria ≥ 1 + and albuminuria A3

	Univariate analysis dipstick ≥ 1+		Multivariable analysis dipstick ≥ 1+		Multivariable analysis of Albuminuria A3	
	**uOR (95 %CI)**	***p*-value**	**aOR (95 %CI)**	***p*-value**	**aOR(95 %CI)**	***p*-value**
Age, years	0.99 (0.98–1.01)	0.815	0.98 (0.98–1.01)	0.909	1.012 (0.98–1.05)	0.317
Sex (male)	1.39 (0.93–2.06)	0.106	1.48 (0.94–2.33)	0.087	2.09 (0.54–8.13)	0.28
Site (rural)	2.98 (1.85–4.80)	< 0.001	1.53 (0.86–2.73)	0.148	3.7 (0.99–13.98)	0.051
Obesity	0.41 (1.49–1.15)	0.091	1.45(0.46–4.52)	0.523	3.54 (0.94–13.38)	0.062
Hypertension	0.93 (0.56–1.55)	0.789	1.03 (0.55–1.95)	0.916	1.00 (0.28–3.63)	0.94
Diabetes	0.69 (0.21–2.29)	0.552	1.36 (0.36–5.11)	0.647	6.12 (1.52–24.53)	0.010
HIV	2.28 (0.25–20.63)	0.462	1.69 (0.15–19.44)	0.674	NA	
Urinary pH	**Ref. 6.5-7.0**					
< 6.5	0.36 (0.98–1.36)	0.134	0.22 (0.04–1.22)	0.084	0.54 (0.09–3.16)	0.501
≥ 7.5	8.76 (4.35–17.61)	< 0.001	7.45 (3.28–16.93)	< 0.001	1.47 (0.37–5.85)	0.578
Urine gravity	**Ref. 1.010–1.020**					
< 1.010	3.90 (2.52–6.04)	< 0.001	2.19 (1.35–3.57)	0.002	1.58 (0.28–8.84)	0.603
≥ 1.020	0.26 (0.11–0.57)	0.001	1.64 (0.50–5.40)	0.650	1.33 (0.25–6.95)	0.734

## Discussion

As part of our population-based study, we here assessed the influence of rural versus urban location on the level and interpretation of kidney markers. We found an overall prevalence of dipstick proteinuria (≥ 1+) of 9.6 %, much higher in the rural (13.1 %) than in the urban (4.8 %) site. Interestingly, the overall prevalence of significant (A2 or A3) albuminuria was 6.6 % and did not differ by site; thus, additional factors must explain the difference observed in dipstick proteinuria between rural and urban subjects. Physical activity, urinary concentration, diet, and urinary pH have previously been associated with the prevalence of dipstick proteinuria [11, 24].

In this study, we found that the frequency of dipstick proteinuria increased when urine was more alkaline (pH ≥ 7.5). Interestingly, after adjustment for age, site, sex and other risk factors, alkaline urine remained independently associated with dipstick proteinuria. Testing for protein by dipstick is based on the protein error of indicators principle. In fact, the reagent area of urine dipstick is impregnated with tetrabromophenol blue indicator buffered to pH 3.0. In the presence of protein (albumin), there is ionization of the indicator (and hence pH) that leads to a change in color from yellow to green, then blue, depending upon the concentration [25]. Thus, alkaline urine may give a false positive result with the reagent strip. However, these interferences are mostly described in vitro, with pH values outside the physiological range [26]. Interestingly certain dietary features are known to be associated with urine pH, in particular a vegetarian diet less enriched in amino acids is associated with alkaline urine, whereas a western diet, typically high in proteins (the most common dietary source of amino acids) is typically associated with acidic urine [27]. Admittedly, we did not collect information about the type of diet of participants, yet the high burden of childhood malnutrition observed in rural areas of DRC is in line with an overall low-protein intake in rural settings [28]. In both sites, dipstick proteinuria was associated with diluted urine at least as assessed by urine specific gravity a finding contrasting with published evidence [24]. However, it should be noted that urine specific gravity is unreliable and much less accurate than osmolality to assess the urine concentration status [29, 30]. Furthermore, a low urine specific gravity may be indicated by urine dipstick tests if the urine is alkaline [25]. Thus, the interpretation of the apparent impact of specific gravity on dipstick positivity remains complex and this further underlines the limitations of the dipstick. Fortunately, in this study, we also measured ACR, which is much less biased by urine hydration status. We confirmed, as shown in previous studies conducted outside SSA [12, 31], that the proteinuria dipstick is inaccurate in detecting albuminuria A2 or A3. For example, 84.6 % of subjects with A2 or 69.2 % with A3 had negative urine dipstick results (false-negative ≥ 1+). In addition, proteinuria dipstick tests had very poor overall sensitivity (< 50 %) for the detection of A2 or A3 in both sites. The low accuracy could be partly explained by the fact that this study was conducted in the general population rather than high-risk subjects, resulting in a low prevalence of proteinuria. Another interesting finding is that the determinants of proteinuria dipstick (≥ 1+) and those of albuminuria (A3) were very distinct. In particular, albuminuria, but not a positive dipstick proteinuria, was significantly associated with diabetes, another reminder of the poor diagnostic value of dipstick. Thus, despite the obvious benefits of proteinuria dipsticks (quick, cheap, and easy) in epidemiologic studies in SSA, researchers need to be aware of its limitations. Nevertheless, it is worth emphasizing that over 70 % of recent general population studies of CKD in SSA still relied on proteinuria dipstick as urinary parameter [2]. Finally, we compared the body composition profile of rural and urban participants, and its influence on eGFR markers. Rural participants had higher skeletal muscle mass, whereas urban residents had higher fat mass, suggesting more physical activity in rural (mostly farmers) participants and/or higher caloric intake in urban participants. The metabolic profile (high BMI, enlarged WC) observed in urban subjects is compatible with the expected effects of Westernization in SSA [3]. Differences in ethnicity could not explain the variability in body composition between rural and urban residents. Indeed the Bashi ethnic subgroup predominates both in Ibanda, the urban site as it does in Katana, the rural site [32]. Despite a higher relative muscle mass in rural subjects, serum creatinine and eGFRcr values did not differ by site, whereas eGFRcys was slightly higher in urban than rural participants, probably due the metabolic profile (high BMI, weight, fat mass) seen in urban compared to rural subjects. Previous findings reported the strong relationship of body mass index and weight with cystatin C level [33, 34]. Further studies are needed in SSA to assess the accuracy of eGFRcr and eGFRcys compared to the measurement of GFR.

### Strengths of the study

The present study included a large representative general population sample. To the best of our knowledge, it is the very first to compare in SSA the assessment of dipstick proteinuria with the gold standard ACR both in rural and urban sites. Although testing for proteinuria by dipstick would decrease costs, it would fail to diagnose most patients with albuminuria, an early marker of kidney disease.

### Limitations of the study

Our study has limitations. First, the estimates were based on single-time measurements rather than on repeat abnormalities over three or more months, as recommended by KDIGO guidelines [23]. This is, however, unfortunately the rule in most large-sized population-based studies. Second, the timing of urine sampling could vary by a few hours between participants, but this did not differ by site. Third, we did not collect information on dietary habits. Fourth, we used visual reading, which is less sensitive than an automated method to detect significant proteinuria [35]. In fact, urine discoloration may cause difficulties for visually interpreting the test results. However, visual reading was performed in both sites, by the same team, using identical procedures, and thus does not account for the differences between sites.

## Conclusions

This study provides a head-to head comparison of dipstick for proteinuria versus the gold standard ACR. Whereas in the urban site, ACR and dipstick had similar positivity rates, in the rural site, dipstick was around 2 times more frequently positive. Although the reasons for this discrepancy are probably multiple, they include a.o. urine pH and specific gravity. Overall, our results call for the use of ACR rather than dipstick in future studies of CKD prevalence in SSA, as it is a more accurate early marker of kidney disease. The expected association of ACR (but not positive dipstick) with diabetes further underscores the major limitations of the dipstick.

## Supplementary Information

**Additional file 1: Supplementary Figure 1. **Flow chart of the study population and data availability. **Supplementary Figure 2. **Correlation between urine specific gravity and osmolality in sub-group of urban (red line) and rural (gray line) subjects. **Supplementary Table 1.** Diagnostic performance of urine dipstick for the detection of ACR ≥ 30 mg/g (A2) and ACR ≥ 300 mg/g (A3). **Supplementary Table 2.** Diagnostic performance of urine dipstick in the study population.

## Data Availability

The datasets used and/or analyzed during the current study are available from the corresponding author on reasonable request.

## Abbreviations

ACR: Albumin-to-creatinine ratio
aOR: Adjusted odds ratio
BMI: Body mass index
CI: Confidence interval
CKD: Chronic kidney disease
CKD-EPI: Chronic kidney disease epidemiology collaboration
CRP: C-reactive protein
Cr: Creatinine
Cys: Cystatine C
DRC: Democratic Republic of Congo
eGFR: Estimated glomerular filtration rate
HC: Hip circumference
HIV: Human immunodeficiency virus
IQR: Interquartile range
KDIGO: Kidney Disease Improving Global Outcome
LBM: Lean body mass
MUAC: Mid-upper arm-circumference
NPV: Negative predictive value
NGOs: Non Governmental organizations
NSAIDs: Non-Steroidal Anti-inflammatory Drugs
PPV: Positive Predictive value
SMM: Skeletal Muscle Mass
SSA: Sub-saharan Africa
TSH: Thyroid stimulating hormone
uOR: Unadjusted Odds ratio
WC: Waist circumference
WHR: Waist to hip ratio
