# Supplementary material for: Impact of rural versus urban setting on kidney markers: a cross-sectional study in South-Kivu, DRCongo
Source: BMC Nephrol. 2021 Jun 25;22:234. doi: 10.1186/s12882-021-02431-w (PMC8229682; doi:10.1186/s12882-021-02431-w)
Supplement: Supplementary file 1 — Additional file 1: Supplementary Figure 1. Flow chart of the study population and data availability. Supplementary Figure 2. Correlation between urine specific gravity and osmolality in sub-group of urban (red line) and rural (gray line) subjects. Supplementary Table 1. Diagnostic performance of urine dipstick for the detection of ACR ≥ 30 mg/g (A2) and ACR ≥ 300 mg/g (A3). Supplementary Table 2. Diagnostic performance of urine dipstick in the study population. [file 12882_2021_2431_MOESM1_ESM.docx]

**Dipstick = 1134**

n= 21 subjects did not give a urine sample,

162 excluded because of the detection of blood, leucocytes and/or nitrites.

**Included n=1317**

n= 730 rural site

n= 587 urban site

**Serum creatinine n=1235**

**eGFRcr n=1235**

**Ratio A/C n=1070**

**Missing = 247 (rural 97, urban 150)**

n= 100 samples lost during transport

n= 126 volume of samples too low

**Cystatin C**

**Missing 128**

n= 82 haemolyzed samples

n= 46 volume of samples too low

**Creatinine**

**Missing n= 82**

-n= 36 haemolyzed samples

-n= 46 volume of samples too low

**Cystatin C n=1189**

**eGFRcr/eGFRcr-cys**

**Excluded n=33**

n= 24 pregnant

n= 9 without consent

**n=1350**

**Population screened**

**Supplementary Figure 1: Flow chart of the study population and data availability.**

**
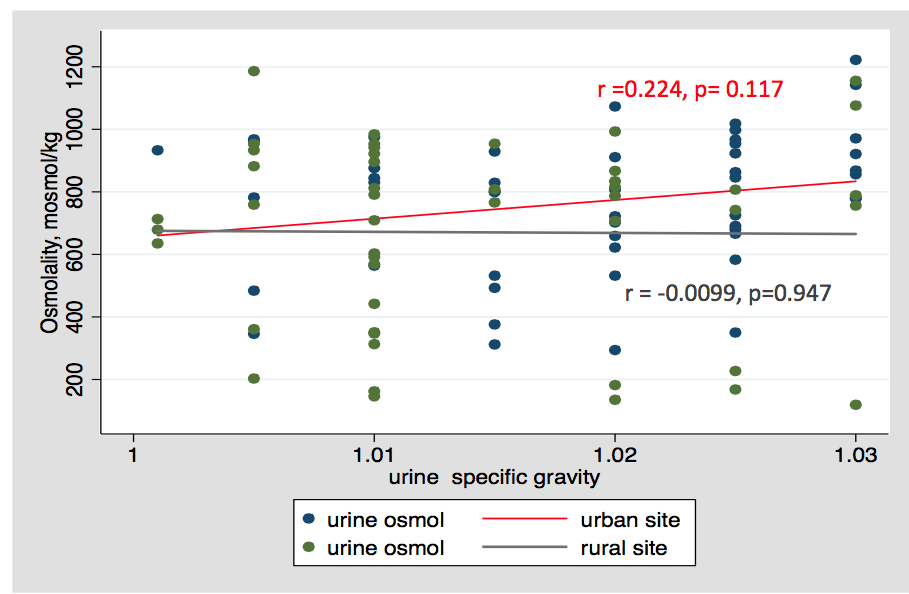
**

**Supplementary Figure 2: correlation between urine specific gravity and osmolality in sub-group of urban (red line) and rural (gray line) subjects.**

**Supplementary Table 1. Positivity of dipstick proteinuria in cases with ACR < 30 mg/g (A1), ≥ 30 mg/g (A2) and ≥ 300 mg/g (A3)**

a) Whole group

|  | ACR (mg/g) | | | Total |
| --- | --- | --- | --- | --- |
| Urine dipstick | < 30 | 30-300 | > 300 |  |
| 0 | 800 | 33 | 9 | 842 |
| ≥ 1+ | 88 | 6 | 4 | 98 |
| Total | 888 | 39 | 13 | 940 |

b) Urban site

|  | ACR (mg/g) | | | Total |
| --- | --- | --- | --- | --- |
| Urine dipstick | < 30 | 30-300 | > 300 |  |
| 0 | 325 | 10 | 8 | 343 |
| ≥ 1+ | 15 | 4 | 1 | 20 |
| Total | 340 | 14 | 9 | 363 |

c) Rural site

|  | ACR (mg/g) | | | Total |
| --- | --- | --- | --- | --- |
| Urine dipstick | < 30 | 30-300 | > 300 |  |
| 0 | 475 | 23 | 1 | 499 |
| ≥ 1+ | 73 | 2 | 3 | 78 |
| Total | 548 | 25 | 4 | 577 |

**Supplementary Table 2. Diagnostic performance of dipstick proteinuria for the detection of ACR ≥ 30 mg/g (A2) and ACR ≥ 300 mg/g (A3)**

1. Whole group

| Standard reference | Cutoff | Sensitivity | Specificity | PPV | NPV |
| --- | --- | --- | --- | --- | --- |
| ACR ≥ 30 mg/g | ≥ 1+ | 19.2 % | 90.1 % | 10.2 % | 95.0% |
| ACR ≥ 300 mg/g | ≥ 1+ | 30.8 % | 89.9 % | 4.1% | 98.9 % |

b) Urban

| Standard reference | Cutoff | Sensitivity | Specificity | PPV | NPV |
| --- | --- | --- | --- | --- | --- |
| ACR ≥ 30 mg/g | ≥ 1+ | 17.2 % | 86.7 % | 25 % | 94.8% |
| ACR ≥ 300 mg/g | ≥ 1+ | 11.1 % | 94.6% | 5 % | 97.7% |

c) Rural

| Standard reference | Cutoff | Sensitivity | Specificity | PPV | NPV |
| --- | --- | --- | --- | --- | --- |
| ACR ≥ 30 mg/g | ≥ 1+ | 21.7 % | 95.6% | 6.4 % | 95.2% |
| ACR ≥ 300 mg/g | ≥ 1+ | 75% | 86.9 % | 3.8 % | 99.8 % |

ACR, spot urine albumin/creatinine ratio (mg/g); NPV, negative predictive value; PPV, positive predictive value.
